# Identification of novel candidate compounds targeting TrkB to induce apoptosis in neuroblastoma

**DOI:** 10.1002/cam4.175

**Published:** 2014-01-01

**Authors:** Yohko Nakamura, Akiko Suganami, Mayu Fukuda, Md Kamrul Hasan, Tomoki Yokochi, Atsushi Takatori, Shunpei Satoh, Tyuji Hoshino, Yutaka Tamura, Akira Nakagawara

**Affiliations:** 1 Division of Biochemistry and Innovative Cancer Therapeutics, Chiba Cancer Center Research InstituteChiba, 260-8717, Japan; 2 Department of Bioinformatics, Graduate School of Medicine, Chiba UniversityChiba, 260-8670, Japan; 3 Department of Physical Chemistry, Graduate School of Pharmaceutical Sciences, Chiba UniversityChiba, 260-8675, Japan

**Keywords:** BDNF, drug discovery, in silico simulations, neuroblastoma, TrkB

## Abstract

Neuroblastoma (NB) is one of the most frequent solid tumors in children and its prognosis is still poor. The neurotrophin receptor TrkB and its ligand brain-derived neurotrophic factor (BDNF) are expressed at high levels in high-risk NBs and are involved in defining the poor prognosis of the patients. However, the TrkB targeting therapy has never been realized in the clinic. We performed an in silico screening procedure utilizing an AutoDock/grid computing technology in order to identify novel small chemical compounds targeting the BDNF-binding domain of TrkB. For the first screening, a library of three million synthetic compounds was screened in silico and was ranked according to the Docking energy. The top-ranked 37 compounds were further functionally screened for cytotoxicity by using NB cell lines. We have finally identified seven compounds that kill NB cells with the IC_50_ values of 0.07–4.6 *μ*mol/L. The terminal deoxynucleotidyl transferase dUTP nick end labeling (TUNEL) assay showed that these molecules induce apoptosis accompanied by p53 activation in NB cell lines. The candidate compounds and BDNF demonstrated an antagonistic effect on cell growth, invasion, and colony formation, possibly suggesting competition at the BDNF-binding site of TrkB. The candidate compounds had tumor-suppressive activity in xenograft and in vivo toxicity tests (oral and intravenous administrations) using mice, and did not show any abnormal signs. Using in silico Docking screening we have found new candidate TrkB inhibitors against high-risk NBs, which could lead to new anti-cancer drugs.

## Introduction

Neuroblastoma (NB) is one of the most common childhood solid tumors occurring from the precursor cells of the sympathoadrenal lineage of the neural crest cells and is responsible for nearly 8% of all pediatric cancers 1. The tumors arise in the peripheral nervous system, such as in the adrenal medulla and in sympathetic ganglia. NBs observed within 1 year after birth often regress spontaneously 2–4. On the other hand, the tumors found in patients over 1 year of age are usually aggressive with poor prognosis, which are frequently associated with chromosomal instability, such as allelic loss of the short arm of chromosome 1, that of the long arm of chromosome 11, gain of chromosome 17q, and amplification of *MYCN* oncogene 5,6. In particular, chromosomal deletions at 1p36 and 11q have been detected in many cancers including NB, and thus the genes located in this region have been the candidates as tumor suppressor genes 5–8.

The Trk (NTRK) family of neurotrophin receptors plays a critical role in development and maintenance of the nervous system. This protein family of tyrosine kinase receptors consists of TrkA (NTRK1), TrkB (NTRK2), and TrkC (NTRK3). Activation of Trk family protein receptors by their preferred neurotrophins (nerve growth factor [NGF] to TrkA, brain-derived neurotrophic factor [BDNF] and NT4/5 to TrkB, and NT3 to TrkC) is closely involved in the survival and differentiation of neurons during development 2–4,9–13. TrkB, one of the members of TRK family of tyrosine kinase receptors mentioned above, is involved in regulating neuronal survival and differentiation 2,4,11. We previously reported that the level of TrkA is significantly higher in favorable NB tissues, while TrkB and its ligands, BDNF and NT4/5, are expressed at high levels in unfavorable NBs and function in an autocrine/paracrine manner to promote cell growth and survival 14. Thus, we hypothesized that a clinical treatment targeting TrkB could improve the prognosis of patients with NB. Toward this end, we performed an in silico screening strategy utilizing grid computing technology (http://www.worldcommunitygrid.org/) to identify novel candidate compounds targeting the BDNF-binding domain of TrkB. The grid-networking system we employed in this project is World Community Gird, which was implemented by the IBM Corporation as a social contribution program. Grid computing technology links many individual computers processing in their spare time, creating a large system with massive computational power far suppressing the power of supercomputers. As the job is split into small pieces that can be processed simultaneously, computation time is reduced from years to days.

In the first screening, a library of synthetic compounds including three million molecules was examined in silico. We further performed in vitro screening assays and identified seven candidate molecules that significantly facilitate growth inhibition in several NB-derived cell lines. We found that these molecules induce apoptosis in NB cell lines at low IC_50_ values, suggesting the molecular mechanism of the cellular growth inhibition. In this study, we demonstrated that novel candidate compounds were rapidly and effectively identified by an in silico Docking screening strategy, followed by in vitro assays. We propose that the candidate compounds targeting the extracellular domain of TrkB could help develop a novel treatment and cure for childhood cancers, including NB.

## Material and Methods

### In silico screening

The three-dimensional structures of the TrkB/BDNF complex on cell membrane were constructed with MOE (version 2009; CCG Inc., Montreal, Canada) and NAMD (http://www.ks.uiuc.edu/Research/namd/) according to the Brookhaven Protein Databank 1WWB.

The molecular mechanistic calculations were performed to obtain the local minimum structure using Amber99 force field in MOE. In silico screening was performed by using AutoDock (http://autodock.scripps.edu/) and the World Community Grid (http://www.worldcommunitygrid.org/). The three-dimensional structures of these complexes were displayed by using MOE.

### Reagents and cell culture

Small molecules were purchased from Namiki Shoji Co. Ltd. (Tokyo, Japan) dissolved in dimethyl sulfoxide (DMSO) at a final concentration of 10 mmol/L, and kept at −20°C. Human NB-derived cell lines were maintained in an RPMI 1640 medium supplemented with 10% heat-inactivated fetal bovine serum at 37°C in a humidified atmosphere of 5% CO_2_ in the air.

### Proliferation assay

The cells were plated in triplicate at a density of 1 × 10^4^ per well in 24-well culture plates. Twenty-four hours after seeding the cells, the cells were treated with small candidate molecules and DMSO as a control at several concentrations (0.1, 1.0, 10 *μ*mol/L) or left untreated. At the indicated time points after the treatment with the small molecules, cells were trypsinized and the number of viable cells was directly scored using a cell counter. Each experiment was performed in triplicate samples.

### TUNEL assay

Cells were grown in a standard culture medium in the presence or absence of the small candidate molecules for indicated periods. Cells were trypsinized, washed with ice-cold phosphate buffered saline (PBS), and then attached to a coverslip using a cytospin centrifuge. Cells were fixed with 4% paraformaldehyde in PBS for 1 h, and permeabilized with 0.1% Triton X-100 and 0.1% sodium citrate in PBS for 2 min. Terminal deoxynucleotidyl transferase dUTP nick end labeling (TUNEL) reaction was performed according to the manufacturer's instructions. The nuclei were stained with DAPI. The coverslips were mounted onto glass slides, and the stained cells were examined by a confocal laser scanning microscope.

### Western blotting

Cells growing in the mid-log phase were washed with PBS and a lysis buffer (10% glycerol, 5% 2-mercaptoethanol, 2.3% sodium dodecyl sulfate (SDS), 62.5 mmol/L Tris-HCl, pH 6.8) was added. Whole cell lysate was subjected to SDS/PAGE (polyacrylamide gel electrophoresis) followed by Western blotting.

### Cellular assays with BDNF treatment

For colony formation assay, SH-SY5Y cells overexpressing TrkB were treated with the candidate compounds (0, 0.1, and 1 *μ*mol/L) in the absence or presence of BDNF (50 ng/mL) for 2 weeks. Images were taken after crystal violet staining. The number and size of colonies were measured in independent triplicate experiments. For the cell invasion assay, SH-SY5Y cells overexpressing TrkB were treated with the candidate compounds in the absence or presence of BDNF (50 ng/mL) for up to 6 days. Cellular invasion was measured by the Boyden chamber method. For phospho-TrkB assay, SH-SY5Y/TrkB cells were incubated in RPMI 1640 medium without serum for 6 h, then pretreated with the medium in the presence or absence of compound G (100 *μ*mol/L) for 5 min. BDNF in the final concentrations of 0, 5, 15, and 25 ng/mL was added, incubated for 10 min, and then whole cell lysate was prepared. TrkB and phosphorylated TrkB proteins were detected by mouse monoclonal anti-TrkB (Z10) antibody (Santa Cruz Biotechnology, Inc., Dallas, TX) and rabbit polyclonal anti-phospho-TrkB (C50F3) antibody (Cell Signaling Technology, Inc., Danvers, MA), respectively.

### Tumor suppression assay utilizing SCID mice

NOD SCID mice (CB-17-Prkdc scid/J) were used for the tumor xenograft experiments. Live animals in this assay were handled, strictly following the protocols and instructions of the Chiba Cancer Center Research Institute, which were approved by our ethics committee. SCID mice were transplanted with CHP134 (5 × 10^6^ cells/100 *μ*L) after 6 weeks of the birth. The compound treatment was initiated at the time when the tumor is about 5 mm^2^. The ratio for the injection (amount of compounds/the weight of mouse) was 2 mg per 1 kg. Candidate compounds were dissolved in DMSO and were diluted with a 20% xylitol solution (10-fold dilution). Each mouse was treated with intraperitoneal injection once a day for 14 days. Tumor volume was measured once every 2 days to evaluate the inhibition capability of the candidate drugs. The length and width of each tumor was measured once in every 2 days to evaluate the inhibition capability of the candidate drugs. Tumor volume was calculated according to the following formula: [length × width^2^]/2 15,16. Animals were treated according to the Institutional Animal Care and Use committee guidelines.

### Statistical analysis

The Mann–Whitney test was used to compare differences between two independent groups of the data in Figures 2, 4, and 6. All the statistical analyses were performed by Excel 2002 (Microsoft, Redmond, WA) with statistics add-in module Statcel2 (OMS publishing Inc., Saitama, Japan). A *P*-value of <0.05 was deemed statistically significant.

## Results

### In silico screening and candidate selection

**Figure 1:**
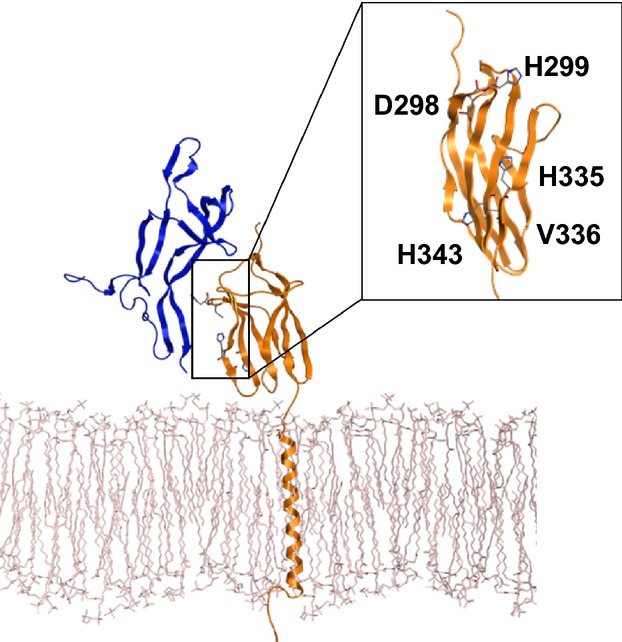
Computational modeling of the specific binding between TrkB (yellow) and BDNF (blue) on the cell membrane (white). 3D structure of TrkB/BDNF on the cell membrane is shown. Inset indicates potential amino acid residues participating in the interaction.

Our goal was to develop novel drugs to treat and cure the patients with high-risk NB. We performed the in silico Docking simulations using AutoDock software to achieve high-throughput screening. World Community Grid (IBM Corp., Armonk, NY) was utilized to accelerate the computations. Because TrkB, one of the neurotrophin (NT) receptors harboring tyrosine kinase activity, is expressed at high levels in aggressive NB 14, we focused on the extracellular BDNF-binding site of TrkB as the target for the in silico screening. According to the molecular simulation of a ternary structure of the TrkB/NT complex, we determined the amino acid residues that participate in the physical interaction between BDNF and TrkB (Fig. 1) 17. We set the docking grid to this BDNF-binding domain of TrkB 18 and performed the in silico screening against a library of three million small chemical compounds as potential drug candidates. Using the docked energy scores given by the AutoDock simulations, the molecular profile of the top 100 molecules was examined in the light of Lipinski's Rule of Five (data not shown) and the highest 60 were chosen for further experiments.

### Candidate compounds inhibit the cellular growth in NB cell lines

**Figure 2:**
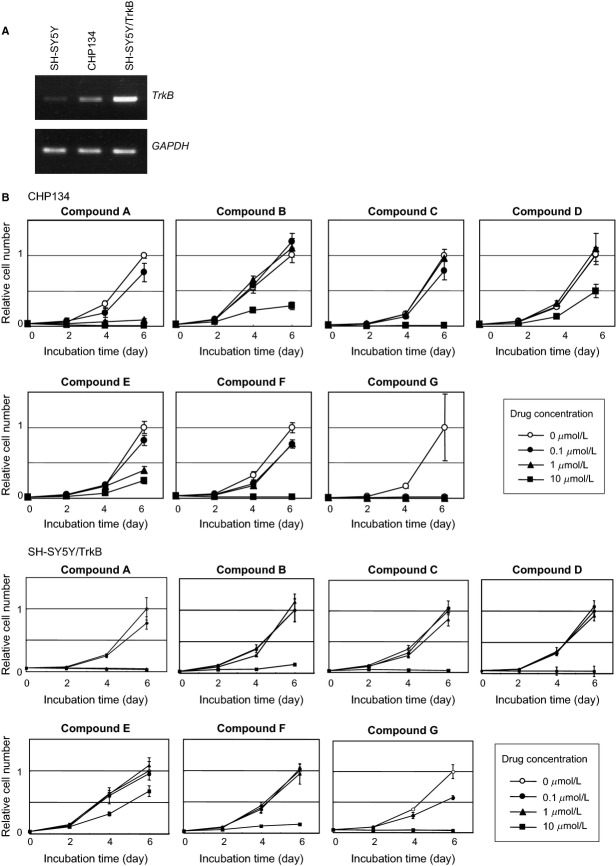
Growth inhibitory effects of compounds A–G in neuroblastoma-derived cell lines. (A) Expression levels of TrkB mRNA in SH-SY5Y, SH-SY5Y/TrkB, and CHP134 cells. SH-SY5Y/TrkB is the cells in which TrkB is stably cloned. (B) Growth curves in the presence or absence of compounds A–G. Compounds concentration: open circle (0 *μ*mol/L), filled circle (0.1 *μ*mol/L), filled triangle (1 *μ*mol/L), filled square (10 *μ*mol/L). The cell numbers were represented as the relative values compared to those without the compounds (open circle) at the time of 6 days (set to 1). The *P*-values between the open circle (0 *μ*mol/L) and filled square (10 *μ*mol/L) at day 6 were less than 0.05 in all experiments using seven compounds.

**Figure 3:**
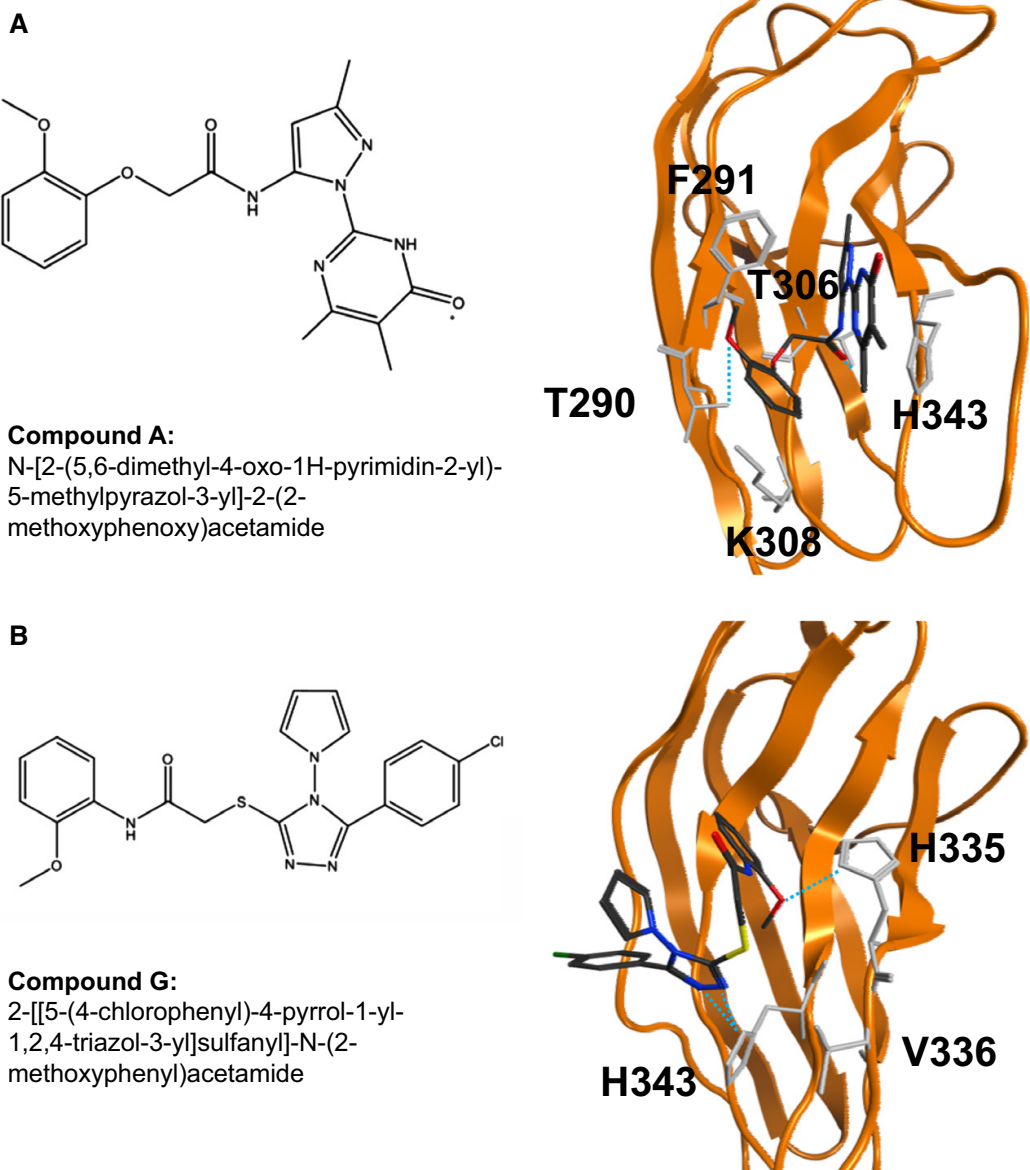
Chemical structure and hypothetical binding model between the molecules A (A) or G (B) and the surrounding residues of TrkB. Amino acid residues involved in the interaction were predicted by the docking simulation.

We expected that the candidate molecules identified above compete with BDNF at the BDNF-binding domain, resulting in inactivation of TrkB activity that triggers the inhibition of the TrkB signaling pathway. As this pathway propagates differentiation and survival signals into the inner part of the cellular fractions, such as the cytosol and the nucleus, endogenous overexpression of the *TrkB* gene frequently found in unfavorable NB may facilitate cellular growth 2,11,12,19,20. Therefore, we examined whether the candidate compounds could decrease the growth rate of NB cell lines in which TrkB expression was either detected (CHP134) or overexpressed (SH-SY5Y/TrkB) (Fig. 2A). Among 60 candidates identified using in silico screening, 37 were available for further screening because 23 chemicals could not be obtained or they could not be dissolved in DMSO. CHP134 cells were treated with a fixed concentration of the candidate compounds and finally seven compounds were selected (Table 1), which yielded the highest toxicity. These seven candidate molecules were designated as compounds A–G. In the second screening, CHP134 cells were treated with increasing concentrations of the candidate compounds and then the number of surviving cells were counted 2, 4, and 6 days after the treatment. We confirmed that the candidate compounds decreased the number of surviving cells at the concentrations of either 1 or 10 *μ*mol/L (Fig. 2B). These results suggest that the seven candidate compounds induce cellular growth inhibition in NB cell line. In addition, in the other NB-derived cell line, SH-SY5Y, in which TrkB was stably overexpressed, essentially similar results were obtained (Fig. 2B). To quantify the cell growth inhibition mediated by the candidate compounds, the value of half maximal inhibitory concentration, namely IC_50_, was evaluated for these seven candidate compounds. In particular, compounds A and G show significantly low IC_50_ values of 0.3 and 0.07 *μ*mol/L, respectively, while the average of IC_50_ values for the seven compounds was 1.9 *μ*mol/L (Table 1). Compounds A and G killed the human foreskin fibroblast cells (strain BJ; ATCC no. CRL2522) at three to five times higher IC_50_ values than NB cell lines (data not shown). On the basis of the chemical structure of compounds A and G (Fig. 3), potential interaction between the compounds and BDNF-binding domain was simulated and this topic is discussed in the Discussion section.

**Table 1:** IC_50_ values of low-molecular-weight compounds in CHP134 and SH-SY5Y/TrkB cell lines.

Compound	IC_50_ in CHP134 (*μ*mol/L)	IC_50_ in SH-SY5Y/TrkB (*μ*mol/L)
Compound-A	0.29	0.3
Compound-B	1.9	2.0
Compound-C	1.4	2.0
Compound-D	4.6	3.5
Compound-E	2.0	2.2
Compound-F	3.5	3.5
Compound-G	0.07	0.18

### Competitive inhibition between candidate compounds and BDNF

**Figure 4:**
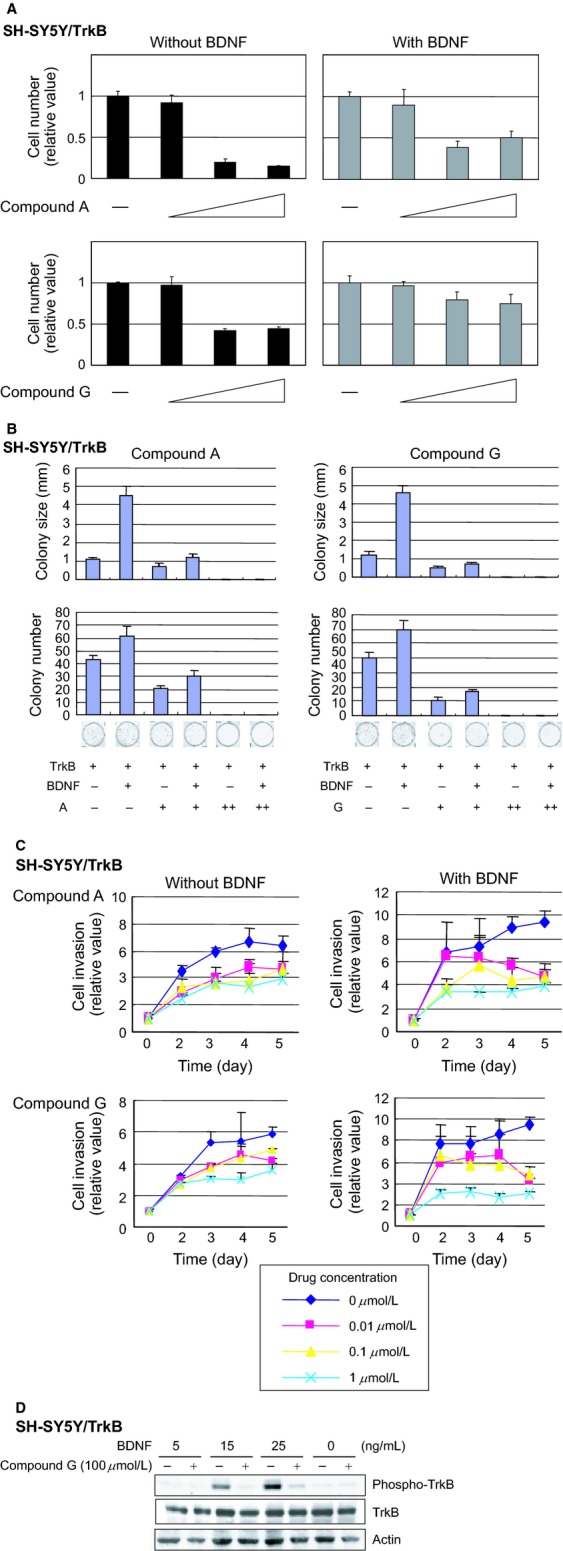
Antagonistic effects of compounds A/G against BDNF in SH-Sy5Y/TrkB cells. (A) Growth inhibitory effect mediated by the compounds A and G in the presence or absence of BDNF. The data plotted relative to the cell number without compound (set to 1). Colony formation assay (B) and cell invasion assay (C) indicating an antagonistic effect between the compounds and BDNF. In the cell invasion assay, the data plotted relative to the value at day 0 (set to 1). The *P*-values between the diamond (0 *μ*mol/L) and cross mark (1 *μ*mol/L) at day 5 were less than 0.05 in all experiments except compound A without BDNF (top left panel). (D) The candidate compounds inhibit phospho-activation of TrkB, which is mediated by BDNF. Cells were pretreated with or without compuond G (100 *μ*mol/L) for 5 min, and were incubated with various concentrations of BDNF (0, 5, 15, 24 ng/mL) for 10 min.

To investigate the relationship between the candidate compounds and BDNF, SH-SY5Y/TrkB cells were treated with compounds in the presence or absence of BDNF. Increasing amount of compound A significantly decreased the number of surviving cells, while the cell growth was partially restored in the presence of BDNF under the same treatment with compound A (Fig. 4A, top panels). This result demonstrates that BDNF partially interferes with the cellular toxicity of compound A, suggesting that compound A and BDNF compete to occupy the BDNF-binding domain of TrkB. Similar competitive inhibition by BDNF was observed in the growth inhibitory effect of compound G (Fig. 4A, bottom panels) and five other compounds (data not shown). The colony formation assay showed that the candidate compounds decrease both colony number and colony size even in the presence of BDNF, indicating that the molecules overcome the cell growth activity of BDNF (Fig. 4B). This result suggests that the compounds and BDNF show an antagonistic effect on cell growth. The cell invasion assay demonstrated that the inhibitory effect on cellular invasion mediated by the candidate molecules was facilitated in the presence of BDNF (Fig. 4C). Furthermore, we examined whether the candidate compounds can inhibit the phospho-activation of TrkB, which was mediated by BDNF. BDNF induces phosphorylation of TrkB in a dose-dependent manner in SH-SY5Y/TrkB cells, while the phosphorylated TrkB was significantly decreased by the addition of compound G in the concentration of 100 *μ*mol/L (Fig. 4D), also supporting the notion above.

### Candidate compounds induce apoptosis in cell lines and inhibit tumor growth in vivo

**Figure 5:**
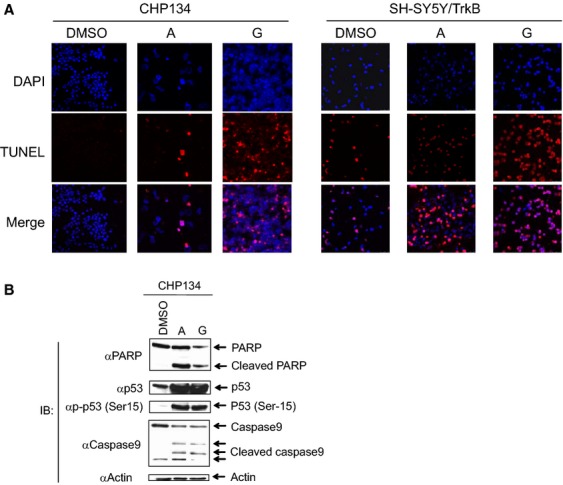
Induction of apoptosis by the candidate compounds. (A) TUNEL assay demonstrating the induction of apoptosis in CHP134 cells. Concentrations of candidate compounds are 5 *μ*mol/L for compounds A and G. Cells were incubated for 3 days prior to TUNEL reaction. DAPI (blue), TUNEL signal (red), and merged images were shown for molecules A and G, respectively. (B) Immunoblotting shows the induction of apoptosis in CHP134 cells. Actin is a loading control.

**Figure 6:**
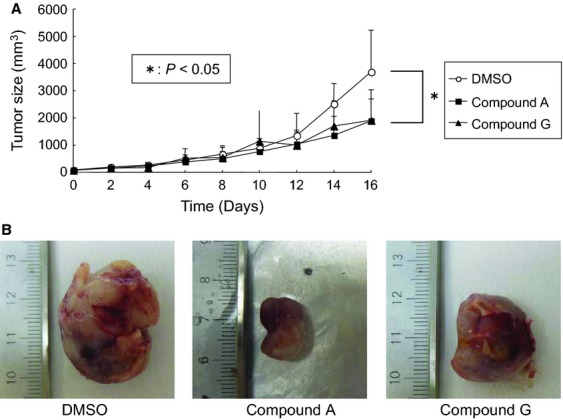
Tumor suppression experiment in vivo utilizing xenografts. (A) When the tumor size reaches to 5 mm square after the injection of CHP134 cells into SCID mice, candidate compounds or mock were treated as described in Material and Methods section. The tumor volume was measured at the indicated days. The data represent the mean value and the error bar represent standard deviation. (B) Representative pictures of tumors obtained at the 20th day after starting administration of the chemicals are shown.

Next, we sought to investigate the possible mechanism of the cellular growth inhibition mediated by the candidate compounds. The treatment of CHP134 cells with optimal concentrations of each candidate compound yielded positive signals in the TUNEL assay (Fig. 5A), which is a typical marker of apoptosis in the cells. The stably cloned strain SH-SY5Y/TrkB also demonstrated similar results. Next, whole cell extracts of CHP134 after treatment with the candidate compounds were analyzed using SDS/PAGE followed by Western blotting (Fig. 5B). The compound treatment induced in the cleavage of PARP and caspase 9. Also, the p53 protein was stabilized and phosphorylated at serine 15. These results strongly suggest that the candidate compounds induce apoptosis in the NB cell line. To further prove the biological activity of the candidate compounds, tumor xenograft studies were performed in SCID mice (Fig. 6). Compounds A and G could significantly decrease the size of tumors in mice compared to xenografts treated with mock (*P* < 0.05), suggesting that the candidate compounds exert antitumor activity in vivo. In contrast, the acute in vivo toxicity test (oral and intravenous administrations) using mice has not shown abnormal signs (data not shown) indicating that the cellular toxicity of the candidate compounds to normal tissue is residual.

## Discussion

In this report, we showed that the candidate molecules of anticancer drug could be screened effectively and rapidly by utilizing an in silico docking approach. We identified several molecules that inhibit cellular growth in NB cell lines. The molecular mechanism of this growth inhibition was the induction of apoptosis accompanied by activation of p53 and caspase 9 as well as PARP cleavage. The candidate compounds demonstrated a competitive inhibitory effect against BDNF, a natural ligand of TrkB.

We generated a possible interaction model between the BDNF-binding site of TrkB and small molecules A and G, which are able to induce cellular growth inhibition in NB 21,22. The docking simulation with molecule A predicted that the amino acid residues of TrkB, such as T290, F291, T306, K308, and H343, presumably participate in the interaction between TrkB and the candidate molecules (Fig. 3A). Among them, H343 is the only residue that is physically located at the BDNF-binding site. Therefore, we presume that the small molecule A could competitively inhibit the binding of BDNF by forming the *π*–*π* interaction with H343. In addition, one of the benzene rings of the molecule A was capable of stabilizing its binding with TrkB by establishing the hydrogen bonds with T290 and T306. Also, the docking simulation with molecule G resulted in the prediction that the residues H335, V336, and H343 of TrkB contribute to the protein-molecule binding (Fig. 3B). In addition, three hydrogen bonds are formed between the molecule G and TrkB (one with H335 and two with H343). We assume that these hydrogen bonds effectively inhibit the interaction between BDNF and TrkB, which may explain the low IC_50_ value of the molecule G.

Because the Trk family of proteins is involved in tumorigenesis, inhibition of tyrosine kinase activity of TrkB has been extensively examined to develop a novel therapeutic treatment in NB 23,24. So far, there is no literature reporting an inhibitor specific to TrkB tyrosine kinase activity. Two types of Trk tyrosine kinase inhibitor have been reported. Cephalon's CEP-751 and CEP-701, namely Lestaurtinib, are structural derivatives of staurosporine and are nonselective tyrosine kinase inhibitors that work on several tyrosine kinases such as TrkA, TrkB, TrkC, and protein kinase C. CEP-751 shows potent inhibitory effect on the enzymatic activity of Trks both in vitro and in intact cells. Interestingly, CEP-751 was also found to be without effect when administered to nude mice bearing SK-OV-3 tumors, which overexpress erbB2 receptors, indicating that inhibition of the Trk receptor tyrosine kinase activity resulted in inhibition of tumor growth 23. A phase 2 study of Lestaurtinib is under way. AZ-23 is another potential molecule that selectively inhibits TrkA/B tyrosine kinase activity 25. In this report, we demonstrated that relatively smaller doses of compounds A and G were required for cell growth inhibition in NB cell lines, compared to necessary doses of existing anti-cancer drugs, as demonstrated by low IC_50_ values. Because the screening of the candidate molecules was performed based on the hypothetical interaction between the candidate molecules and the BDNF-binding domain rather than the tyrosine kinase domain of TrkB, the results of low-dose effect could be explained as follows: TrkB is a transmembrane protein and the BDNF-binding domain is located outside of the cellular membrane, whereas the tyrosine kinase domain is located inside the cell. Thus, it is not necessary to bring the candidate drug interaction with the BDNF domain into the inner part of the cell. Consequently, it is speculated that highly efficient molecules were identified by our in silico approach.

The candidate compounds were screened for the molecules that interact with BDNF-binding domain of TrkB. Our results showed that the molecules and BDNF may competitively bind to the domain. Therefore, we first expected that the molecules simply inhibit the cell growth without apoptosis, since BDNF triggers the signal for cell growth, survival and differentiation. In fact, the inhibitor of the intracellular kinase domain of TrkB does not induce apoptosis. However, we unexpectedly found that the candidate compounds, which are competitors to BDNF, facilitate apoptosis. We speculate that the interaction between the compounds and TrkB may induce a change in the ternary structure of the inner cellular domain of TrkB, which may participate in the regulation of apoptosis. TrkB, which initiates the survival signal during neural development, may play a conflicting role such as regulating tumor suppressive functions, which are driven by apoptosis. As TrkB is also reported to be involved in regulating tumor invasion and metastasis in some adult cancers 26–28, these candidate compounds might also be used as drugs against the other advanced cancers with expression of TrkB.
